# Transcriptome-wide signatures of tumor stage in kidney renal clear cell carcinoma: connecting copy number variation, methylation and transcription factor activity

**DOI:** 10.1186/s13073-014-0117-z

**Published:** 2014-12-11

**Authors:** Qi Liu, Pei-Fang Su, Shilin Zhao, Yu Shyr

**Affiliations:** Center for Quantitative Sciences, Vanderbilt University School of Medicine, Nashville, TN 37232 USA; Department of Biomedical Informatics, Vanderbilt University School of Medicine, Nashville, TN 37232 USA; Department of Statistics, National Cheng Kung University, Tainan, 70101 Taiwan; Department of Cancer Biology, Vanderbilt University School of Medicine, Nashville, TN 37232 USA; Department of Biostatistics, Vanderbilt University School of Medicine, Nashville, TN 37232 USA; School of Life Sciences & Biotechnology, Shanghai Jiao Tong University, Shanghai, 200240 China

## Abstract

**Background:**

Comparative analysis of expression profiles between early and late stage cancers can help to understand cancer progression and metastasis mechanisms and to predict the clinical aggressiveness of cancer. The observed stage-dependent expression changes can be explained by genetic and epigenetic alterations as well as transcription dysregulation. Unlike genetic and epigenetic alterations, however, activity changes of transcription factors, generally occurring at the post-transcriptional or post-translational level, are hard to detect and quantify.

**Methods:**

Here we developed a statistical framework to infer the activity changes of transcription factors by simultaneously taking into account the contributions of genetic and epigenetic alterations to mRNA expression variations.

**Results:**

Applied to kidney renal clear cell carcinoma (KIRC), the model underscored the role of methylation as a significant contributor to stage-dependent expression alterations and identified key transcription factors as potential drivers of cancer progression.

**Conclusions:**

Integrating copy number, methylation, and transcription factor activity signatures to explain stage-dependent expression alterations presented a precise and comprehensive view on the underlying mechanisms during KIRC progression.

**Electronic supplementary material:**

The online version of this article (doi:10.1186/s13073-014-0117-z) contains supplementary material, which is available to authorized users.

## Background

It is now widely accepted that cancer develops through a series of stages [1]. In the early stage, cancer cells, confined to a very limited area, are not invasive and metastatic, whereas in the late stage, the cells, spreading to distant sites in the body, are highly invasive and metastatic. Comparative analysis of expression profiles between the early and late stages of cancers has identified genes with stage-dependent expression alterations, most of which have potential function in inducing and suppressing cancer metastasis [2–6]. These findings help to get a better understanding of cancer progression and metastasis and to predict the clinical aggressiveness of cancer. However, the mechanisms that give rise to these expression alterations remain largely unknown.

An altered transcriptional regulatory network is one major cause for the dysregulated expression during cancer progression, mainly due to activity changes in transcription factors (TFs). The determination of TF activity is difficult since it is generally regulated at the protein level and thus undetectable by transcription profiling. Much effort has been given to using reverse-engineering techniques to infer TF activity, which is responsible for differential expression across conditions [7–14]. These techniques combine TF binding site information with expression profiles to distinguish active TFs from inactive TFs. Recently, similar techniques have been extended and applied to breast cancer and leukemia, helping us identify important TFs in disease development [15–18]. For example, Cheng *et al*. [15] developed a method called BASE to infer TF activity in tumor samples by integrating expression data and TF binding sites (positional weight matrix from the TRANSFAC) and then investigated the correlation between activity profiles and patient survival. They found ATF/CREB and TAL1 were significantly correlated with breast cancer and acute myeloid leukemia patient survival, respectively. As another example, Zhu *et al*. [16] proposed REACTIN to reveal the activity changes of TFs between disease and normal samples. Combining expression data with ChIP-seq data from ENCODE [19], REACTIN successfully detected activity changes of estrogen receptor between estrogen receptor-positive and negative samples in breast cancer. However, the activity changes of TFs are not the only factor responsible for aberrant transcriptional profiles. Other genetic and epigenetic alterations, such as DNA copy number or CpG island methylation, also contribute to gene expression variations [20]. Systematic modeling of transcriptional regulatory programs, which accounts for gene expression alterations beyond genetic and epigenetic contributions, will provide a more accurate and powerful way to elucidate the relationship between TFs and disease.

**Figure 1:**
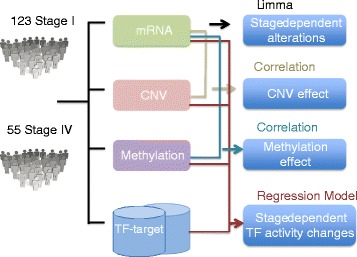
**Flowchart of methods.** We first identified stage-dependent gene expression alterations, then evaluated the effect of CNV/methylation on expression, and finally built a model to infer stage-dependent activity changes of transcription factors by combining the contributions of CNV/methylation.

Large-scale cancer genomics projects such as TCGA (The Cancer Genome Atlas Research Network) are currently generating multiple layers of genomics data for each tumor, including DNA copy number, methylation, and mRNA expression, which provide a great opportunity for systematic modeling of dysregulated transcription. Here, we first identified differentially expressed genes between 123 stage I and 55 stage IV kidney renal clear cell carcinoma (KIRC) patients. Then, we demonstrated contributions of copy number variation (CNV) and methylation variation to gene expression alterations by calculating the correlation of CNV/methylation with expression of all genes and genes with stage-dependent alterations. Finally, we propose a multivariate regression model to infer TF activity changes by associating gene expression outputs with TF binding events beyond the effect of copy number and DNA methylation across KIRC stages (Figure 1). Unlike the recent integrative method modeling the general impact of copy number alterations on gene expression changes [20], our approach models the gene-specific contributions of both copy number and methylation to mRNA expression. The model shows improved prediction performance, further demonstrates the role of methylation as a significant contributor to stage-dependent expression alterations, and identifies key TFs as potential drivers of cancer progression. Dissecting the effect of copy number, methylation and TF activity changes on each individual gene with stage-dependent expression alteration gives a more comprehensive view of underlying mechanisms.

## Methods

### Data and preprocessing

CNV, DNA methylation, mRNA expression profiles and clinical information for KIRC patients were downloaded from the Broad Institute’s Genome Data Analysis Center [21]. In total, 178 common samples with CNV, methylation and RNA-seq data were available, including from 123 stage I and 55 stage IV patients (Table 1). RSEM, based on a general probabilistic model of maximum expectation, was used to estimate gene expression abundance [22]. To determine whether there were problematic samples, we calculated the expression similarity between samples and checked the percentage of necrotic cells, normal cells, and tumor nuclei. Samples with different expression profiles from others were composed of at least 85% tumor nuclei, less than 5% normal cells, or less than 15% necrotic cells (Additional file 1). Therefore, we kept all 178 samples for downstream analysis. Pearson correlations between mRNA expression and CNV/methylation were calculated. Gene set enrichment analysis (GSEA) [23] was used to determine functions significantly associated with high or low correlations between mRNA expression and CNV/methylation. TF binding information was downloaded from MsigDB, which strengthened the prediction of true binding sites by considering the conservation across genomes [24]. After TFs with the same target sets were combined, 206 TFs were left. Among 14,555 genes with matched mRNA, methylation, and CNV data, 5,684 genes had more than 5 interacting TFs.

**Table 1:** **Characteristics of patients with RNA-seq, copy number variation and methylation data for kidney renal clear cell carcinoma**

	**Stage I**	**Stage IV**
	**(n = 123)**	**(n = 55)**
Age in years (mean ± standard deviation)	59.5 ± 13.1	62.8 ± 9.1
Gender, male; n (%)	73 (59.3%)	39 (70.9%)
Median follow-up in months (minimum - maximum)	7.3 (0.03-14.8)	10.1 (0.03-14.9)
Number of deaths (%)	17 (13.8%)	43 (78.2%)

### Stage-dependent expression alterations

Limma [25] was applied to identify differentially expressed genes between 123 stage I and 55 stage IV patients using the following criteria: (1) fold change (FC) >2; and (2) false discovery rate (FDR) <0.001 (Benjamini and Hochberg’s multiple-test adjustment). Functional enrichment analysis on the up-regulated and down-regulated genes was implemented separately in Gene Ontology biological process as well as KEGG (Kyoto Encyclopedia of Genes and Genomes) pathways by WebGestalt [26,27]. Enrichment *P*-values were generated by a hypergeometric test and adjusted by Benjamini and Hochberg’s multiple-test [28].

### A multivariate regression model to connect copy number variation, methylation and transcription factor activities

We associated gene expression outputs with TF binding events beyond the effect of copy numbers and DNA methylation to infer TF activity changes across KIRC stages. Assuming gene expression alterations were due to a simple linear sum of activity changes in bound TFs and copy numbers and DNA methylation variations, we built a multivariate regression model where the dependent variable is the log expression of genes, while independent variables consist of copy numbers, DNA methylation and predicted TF binding sites (Equation 1):

$$ {y}_{ij}={\beta}_i^{CN}{C}_{ij}+{\beta}_i^{Me}{M}_{ij}+{\displaystyle \sum_f}{\beta}_f{S}_j{B}_{fi} $$

1 $$ \begin{array}{l}{S}_j=\left\{\begin{array}{c}\hfill 1,\ j\in stage\ IV\hfill \\ {}\hfill 0,\ j\in stage\ I\ \hfill \end{array}\ {B}_{fi}\right.=\left\{\begin{array}{c}\hfill 1,\ f\  binds\ to\  gene\ i\hfill \\ {}\hfill 0,\  otherwise\ \hfill \end{array}\right.\ \\ {}\end{array} $$

where *y*_*ij*_, *C*_*ij*_ and *M*_*ij*_ represent the log mRNA expression, copy number, and DNA methylation of gene *i* in sample *j*, while *S*_*j*_ denotes the stage information of sample *j* and *B*_*fi*_ suggests whether TF *f* binds to gene *i*. The regression coefficients ${\beta}_i^{CN}$ and ${\beta}_i^{Me}$ estimate the contribution of copy numbers and methylation to mRNA expression for gene *i*, while *β*_*f*_ infers the activity change of TF *f* in stage IV versus stage I. These regression coefficients were determined by minimizing the sum of squared residuals, defined as $SSE={\displaystyle \sum_{ij}}{\left({y}_{ij}^{observed}-{y}_{ij}^{predicted}\right)}^2.$

The expression abundances of 5,684 genes across 178 samples were used to infer TF activity changes across stages. That is, the number of data points n is 1,011,752 (5,684 × 178), while the number of regression coefficients p is 11,574 (206 + 5,684 × 2). The solution is unique since p < n. In addition, residuals look randomly scattered around 0, and there is no evidence of a nonlinear pattern, which suggests the linear regression model is a good fit to the data (Additional file 2). We also used lasso and ridge regression to model the expression changes and obtained similar results (Additional file 3).

## Results

### Stage-dependent expression alterations

We identified 279 differentially expressed genes with FC >2 and FDR <0.001. Of these, 178 (63.8%) had significantly increased abundances, and 101 genes (36.2%) had reduced expression in stage IV versus stage I cancer (Additional file 4).

**Figure 2:**
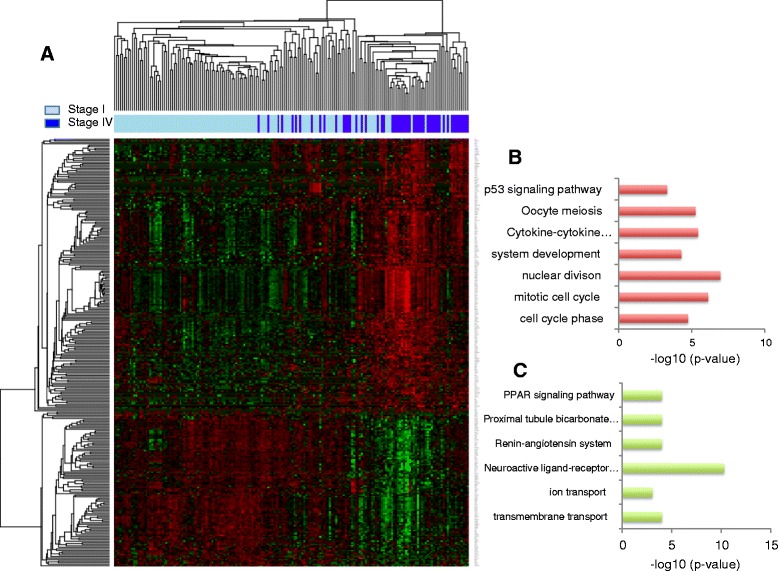
**Stage-dependent transcriptome signatures. (A)** Hierarchical clustering of 123 stage I and 55 stage IV KIRC cancer patients based on 279 stage-dependent gene expression signatures. Red indicates upregulated and green indicates downregulated genes in stage IV. **(B)** Functions enriched in upregulated genes. **(C)** Functions enriched in downregulated genes.

A clear difference between expression profiles of early and late stage cancers was demonstrated in a heat map, using the 279 differentially expressed genes (Figure 2A). The up/down-regulated genes were further interpreted in the context of Gene Ontology biological process as well as KEGG pathways (Additional file 4). Cell cycle (*P* = 1.9e-05), nuclear division (*P* = 1. 1e-07), system development (*P* = 5.2e-05), cytokine-cytokine receptor interaction (*P* = 3.85e-06), and p53 signaling pathway (*P* = 5.0e-04) are significantly enriched in the up-regulated genes, while PPAR signaling pathway (*P* = 1.0e-04), ion transport (*P* = 9.0e-04), transmembrane transport (*P* = 1.0e-04) and neuroactive ligand-receptor interaction (*P* = 4.93e-11) are enriched in the down-regulated genes (Figure 2B). Most of the pathways are involved in tumor growth, invasion, and metastasis, which is consistent with our existing knowledge of cancer progression. Notably, cytokine and cytokine receptor interactions play crucial roles in cancer development and progression [29,30]. Ten genes involved in this pathway were up-regulated, including *CXCL13*, *XCL2*, *XCL1*, *IL2*, *AMH*, *LTB*, *INHBE*, *TNFRSF18*, *CSF2* and *IFNG*. CXCL13 has been implicated in the progression of breast cancer [31], and the addition of lymphotactin (XCL1 and XCL2) has been shown to stimulate ovarian cancer cell migration and proliferation [32]. Cell cycle with 26 genes significantly up-regulated in late stage cancer is also a well-known pathway involved in tumor progression. Among these 26 genes, some have already demonstrated their function in the progression of other types of cancer - for example, CCNA1 [33,34] and CDC20 [35]. In the p53 signaling pathway, *RRM2*, *GTSE1*, *BAI1* and *CCNB2* were significantly overexpressed in the late stage KIRC patients. The depletion of RRM2 has been reported to inhibit tumor growth in head and neck, lung, and ovarian cancers [36,37]. The increased GTSE1 correlates with the invasive potential of breast cancers [38]. Their increased expression in the late stage and the fact that they contribute to the progression and invasion of other types of tumors indicates the important roles of these genes in the progression of KIRC.

PPAR signaling pathways, significantly enriched in down-regulated genes, are responsible for the regulation of cellular events ranging from glucose and lipid homeostasis to cell differentiation and apoptosis. Additionally, emerging evidence indicates their anti-proliferative actions or tumor-promoting effects [39].

### Contributions of copy number variation and methylation to modulation of gene expression

**Figure 3:**
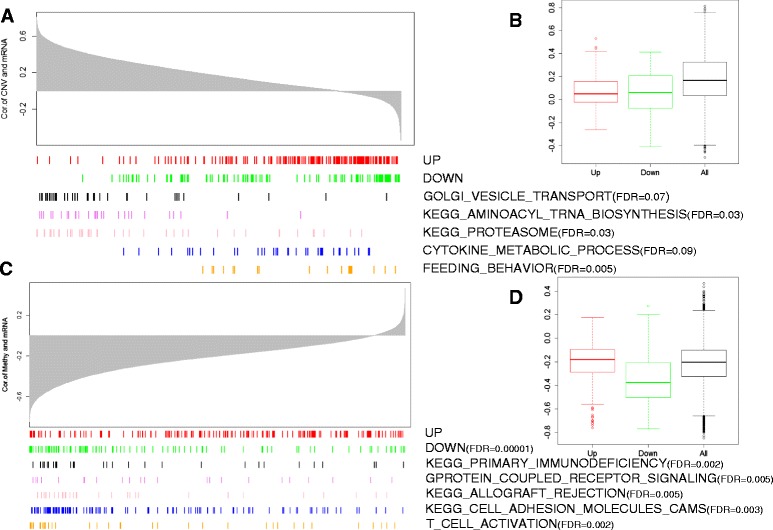
**The effects of CNV and methylation on gene expression. (A)** Ranked correlations of mRNA expression and CNV (top). The locations of upregulated and downregulated genes and genes in the significantly associated functions are shown in the ranked list (bottom). **(B)** The distributions of correlations between mRNA expression and CNV for upregulated genes, downregulated genes and all genes. **(C)** Ranked correlations of mRNA expression and methylation (top). The locations of upregulated and downregulated genes and genes in the significantly associated functions are shown in the ranked list (bottom). **(D)** The distributions of correlations between mRNA and methylation for upregulated genes, downregulated genes and all genes.

To evaluate the contribution of CNV and methylation to the modulation of stage-dependent gene expression alterations, we measured the quantitative relationships between CNV/methylation and mRNA expression abundances using Pearson correlation coefficients. A strong positive correlation was observed between CNV and expression with a median correlation coefficient of 0.17, which is consistent with the role of CNV in modulating gene expression (Figure 2A). Out of 13,508 genes, 10,989 (81.3%) showed positive correlations, of which 5,994 (44.3%) had significant correlations (*P* < 0.01) between CNV and expression, while only 2,519 (18.6%) showed negative correlations, of which 227 (1.68%) had significant correlations (Additional file 5). GSEA [23] showed that positive correlations were represented by Golgi vesicle transport, aminoacyl-tRNA biosynthesis, and proteasome, while negative correlations were represented by cytokine metabolic process and feeding behavior (Figure 3A). In contrast, genes with significant stage-dependent expression alterations did not show strong positive correlations between CNV and expression (median = 0.03). Those up/down-regulated genes were not enriched in the positive correlations (Figure 3A). Out of 178 differentially expressed genes in late stage versus early stage cancer, only 46 genes (25.8%) showed significant positive correlations. The correlation coefficients between CNV and expression abundances of differentially expressed genes were even lower than for other genes (Figure 3B), suggesting that CNV is not a major factor in driving expression alterations during KIRC cancer progression. However, CNV has important effects on expression changes of some cancer-related genes. *FOXM1* (FC = 1, FDR = 6.7e-06) and *CDCA3* (FC = 1.2, FDR = 6.3e-07) are two representative genes overexpressed in late stage cancer, which also had high correlation coefficients between CNV and expression level (R = 0.70 and R = 0.54, respectively).

A strong negative correlation was observed between DNA methylation and expression with a median value of -0.20, which is consistent with the role of methylation in repression of gene expression (Figure 3C). Out of 13,982 genes, 12,629 (90.3%) showed negative correlations, of which 6,552 (46.9%) were significant (*P* < 0.01), while only 1,353 (9.68%) showed positive correlations, of which 47 (0.33%) were significant (*P* < 0.01) (Additional file 6). GSEA [23] showed that negative correlations were represented by primary immunodeficiency, G-protein-coupled receptor signaling pathway, allograft rejection, cell adhesion molecules (CAMs), and so on (Figure 3C). Notably, genes down-regulated in late stage cancer were enriched in negative correlations (FDR = 1.0e-05) with a median value of -0.37. Among 101 down-regulated genes, 92 (91.1%) showed negative correlations and 67 (66.3%) were significant. Compared with up-regulated genes and other genes, DNA methylation was more negatively correlated with expression abundances for down-regulated genes (*P* < 2.2e-16; Figure 3D), suggesting that methylation is a major cause leading to decreased expression abundance during cancer progression. More interestingly, the most negatively correlated gene, *AQP1* (R = -0.77, FC = -1.18, FDR = 1.54e-05), has been shown to be related to tumor progression [40] and high *AQP1* expression has been demonstrated to be associated with better prognosis and improved overall survival outcome in renal tumor patients [41].

### Expression alterations explained and transcription factor activity changes inferred by the model

Since CNV and methylation modulate gene expression, connecting these influential factors in the model is expected to give a comprehensive view of the underlying mechanisms of stage-dependent expression alterations and to provide more power for inferring transcriptional programs driving tumor progression. Here we built a multivariate regression model to infer activity changes of TFs beyond copy number and methylation status variations using TF binding sites as features (see Methods).

**Figure 4:**
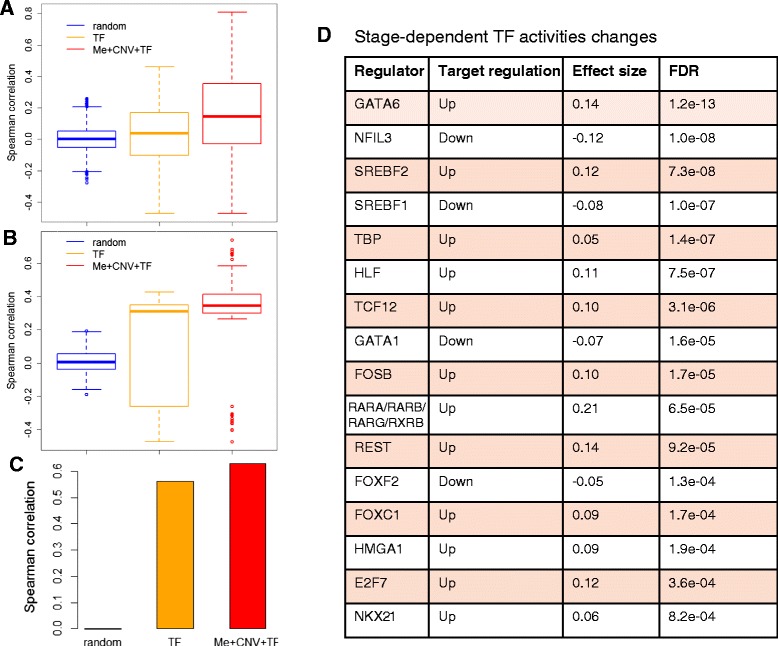
**The integrative model predicts expression alterations and TF activity changes. (A,B)** The distribution of Spearman correlations of observed and predicted gene expression for all genes **(A)** and differentially expressed genes **(B)**. The TF-only model (orange) is significantly better than random (blue, *P* < 2.2e-16), while the integrative model combining TFs, CNV and methylation (red) is significantly better than the TF only model (*P* < 2.2e-16). **(C)** Spearman correlations of observed and predicted expression changes for differentially expressed genes using random, TF-only, and the integrative model connecting TFs, CNV and methylation. **(D)** Candidate regulators associated with KIRC stage with effect size >0.5 and FDR <0.001.

We first assessed whether the integrative model could be trained to predict gene expression abundances across samples. In a 10-fold cross-validation experiment on held out patients and genes, we obtained a mean Spearman rank correlation between predicted and observed gene expression abundance of 0.16 (median = 0.14). By contrast, if we only consider transcriptional effect without taking genomic and epigenomic contributions into account (the TF-only model), we obtained a mean Spearman rank correlation of 0.03 (median = 0.04). Furthermore, if we randomized gene expression abundances and trained the integrative model, we obtained a mean Spearman rank correlation just around 0 (mean = 0.0007, median = 0.001; Figure 4A). For the 279 differentially expressed genes, our integrative model predicted gene expression accurately with a mean Spearman correlation of 0.28 (median = 0.35), while the TF-only model obtained a mean Spearman correlation of 0.16 (median = 0.31) (Figure 4B; Pearson correlation had similar results). We detected expression changes in the late stage compared with the early stage using the predicted expression abundances and found that the predicted log expression changes across stages were highly correlated with the observed ones (Spearman correlation = 0.63). By contrast, the TF-only model obtained a modest correlation between predicted log expression changes and the observed changes (Spearman correlation = 0.56), and the random model failed to predict stage-dependent gene expression changes with a Spearman correlation around 0 (Figure 4C). The significant improvement of the integrative model (*P* < 2.2e-16, Kolmogorov-Smirnov test) further underscores the important role of CNV and methylation on stage-dependent gene expression alterations.

Figure 4D summarizes the predicted dysregulation of 16 TFs in late stage cancer with FDR <0.001and effect size >0.5 (Additional file 7). Many TFs are well-known regulators in tumor progression and metastasis. The most significant TF, GATA6, has been reported to promote colorectal cancer invasion [42], and its aberrant expression is correlated with poor prognosis and liver metastasis in colorectal cancer [43]. The second TF, NFIL3, restricts expression of certain FOXO targets and its expression in cancer is associated with patient survival [44]. A correlation of TCF12 overexpression with colorectal cancer metastasis has also been suggested and validated [45].

Among the 16 TFs, 5 (HLF, E2F7, HMGA1, REST, and FOSB) were significantly changed at the mRNA expression level (Additional file 4). Furthermore, the predicted target regulation of TFs matched the direction of mRNA expression alterations and the function of TFs. For example, REST was down-regulated in the late stage (log_2_(FC) = -0.45, FDR = 0.005), which agreed with the predicted up-regulation of its target genes since REST encodes a transcriptional repressor. As another example, HMGA1, reported to function as a transcriptional mu enhancer, was up-regulated at the mRNA expression level (log_2_(FC) = 0.72, FDR = 0.002), and its target genes were consistently predicted to be up-regulated. For some TFs with context-dependent function, our prediction helped to determine their role in late stage KIRC. For example, E2F7 mainly acts as a transcription repressor [46,47] but activates expression of the *VEGFA* gene when associated with HIF1A [48]. Here, E2F7 was up-regulated in the late stage (log_2_(FC) = 0.95, FDR = 1.1e-04), and its target genes were also up-regulated, which implies that E2F7 might function as a transcriptional activator in late stage KIRC.

The remaining 11 TFs did not show significant mRNA expression changes in late stage versus early stage cancer. The main reason for this is that the ability of TFs is generally modulated at the post-transcriptional and post-translational levels, which will affect TF activity without changing their mRNA abundance.

**Figure 5:**
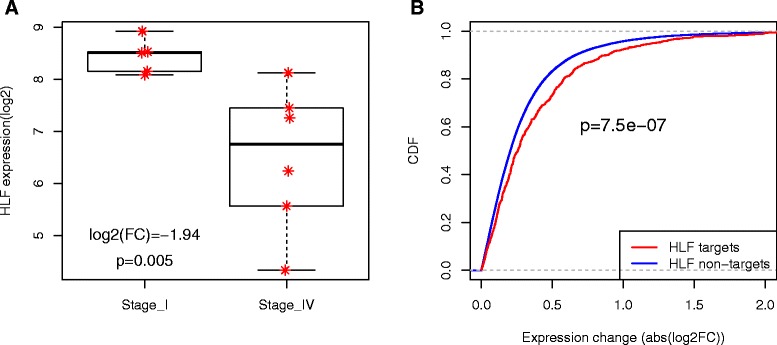
**Activity change of HLF in stage IV versus stage I in the GSE36895 dataset. (A)** HLF is downregulated in stage IV versus stage I in the GSE36895 dataset. **(B)** Expression of HLF targets is more likely to be changed compared with non-targets.

We further validated the stage-dependent TF activity changes using the GSE36895 dataset from the Gene Expression Omnibus, which includes 29 KIRC tumors with stage information. Despite a very small sample size (five stage I and six stage IV patients), HLF was found to be significantly down-regulated in the stage IV versus stage I patients (log2(FC) = -1.94, *P =* 0.005; Figure 5A), which is consistent with our results from the TCGA dataset. Furthermore, the expression of HLF targets was more significantly changed across stages than other non-targets (*P* < 1e-06, one sided Kolmogorov-Smirnov test; Figure 5B). These results support the stage-dependent activity changes of HLF and also indicate HLF changes its activity through mRNA expression alteration. Although the remaining 15 TFs did not show stage-dependent expression changes in the GSE36895 dataset, the expression of their targets was more likely to be changed in the late versus the early stages than non-targets except for TCF12, REST and E2F7, which provided indirect evidence of activity changes of these TFs (Additional file 8).

### Dissecting the role of transcription factor activity, copy number variation and methylation

**Figure 6:**
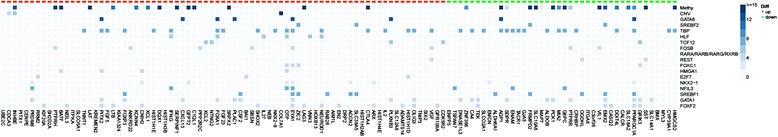
**The effect of CNV, methylation and TF activity on stage-dependent alteration of gene expression.**

**Figure 7:**
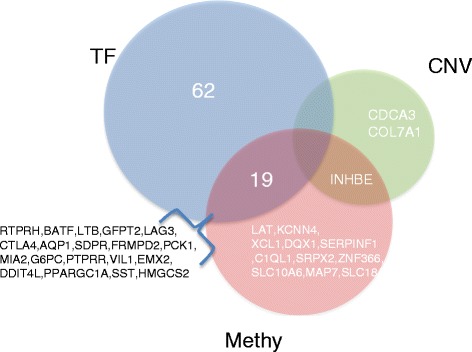
**Venn diagram of genes whose stage-dependent expression alteration is regulated by TFs, CNV and DNA methylation.**

Among 117 differentially expressed genes with known TF binding information, 81% (95) of gene expression alterations can be partially explained by changes in DNA methylation, copy number, or TF activities (Additional file 9). Methylation status changes were involved in alteration of expression of 31 genes and TF activity changes in 81 genes, while CNV was the possible cause of altered expression in only 3 genes (*CDCA3*, *INHB3*, and *COL7A1*) (Figures 6 and 7). These results further demonstrate that methylation and TF activity changes had a major effect on stage-dependent expression alterations compared with CNV.

Dissecting the specific roles of TF activity, CNV, and methylation status changes on individual gene expression alterations provides a more precise view of the underlying regulatory mechanism (Figure 6). For example, *CDCA3* was overexpressed in stage IV cancer (log_2_(FC) = 1.2, FDR = 6.39e-07) and overexpression of *CDCA3* has been reported to be associated with oral cancer progression [49] and prostate cancer [50], which suggests that *CDCA3* also plays an important role in KIRC progression and serves as a potential therapeutic target for KIRC. Our model revealed that the overexpression of *CDCA3* was mainly due to gene amplifications. *XCL1* (log_2_(FC) = 1.33, FDR = 8.71e-05) and *SRPX2* (log_2_(FC) = 1.45, FDR = 0.0002) have been reported to enhance cancer progression and promote cancer migration [32,51], the up-regulation of which was mainly caused by de-methylation in the stage IV cancer. In contrast, the overexpression of *INHBE* (log_2_(FC) = 2.13, FDR = 7.90e-07) was caused by both gene amplification and promoter demethylation. *SLC14A1* and *TEK*, underexpressed in the stage IV cancer (log_2_(FC) = -1.06, FDR = 0.0007; log_2_(FC) = -1.09, FDR = 9.26e-06), were two genes only regulated by GATA1. Consistently, previous studies have demonstrated the down-regulation of these two genes after GATA1 knockdown [52,53]. Since these genes with stage-dependent expression alterations were highly associated with tumor progression and metastasis, the precise view of the underlying regulatory mechanism would be helpful for guiding a future potentially successful novel therapeutic target discovery and eventual use as a patient stratification guide for cancer treatment.

## Discussion

We present an integrative model connecting copy number, methylation, and TF activities to explain genome-wide stage-dependent transcriptome signatures in cancer. The model predicts gene expression abundances accurately and successfully identifies TFs responsible for stage-dependent expression alterations. Dissecting the role of CNV, altered methylation, and TF activity changes on individual gene expression alterations in late stage versus early stage cancer provides new insight into the molecular mechanisms driving tumor progression. To our knowledge, this is the first time that gene-specific contributions of both CNV and methylation have been used to model the transcriptional regulation effect.

An interesting observation is that genes down-regulated in the late stage show higher inverse correlation between DNA methylation and expression abundance than other genes, which suggests down-regulated expression is partly due to altered DNA methylation (see the ‘Contributions of copy number variation and methylation to modulation of gene expression’ section above). This finding is consistent with previous studies that have reported accumulation of DNA methylation changes across tumor stages and an increase of promoter methylation levels of cancer-related genes during cancer progression [54–61]. Specifically, tumor progression has been shown to be characterized by global DNA hypomethylation in the early stage of carcinogenesis and locus-specific DNA hypermethylation predominantly in the late stage in the transgenic adenocarcinoma of mouse prostate model (TRAMP) [55,62]. Our findings also demonstrated the necessity of building an integrative model to take into account all potential factors modulating gene expression and identify TFs associated with cancer progression in a more accurate and powerful way.

There are many opportunities to improve the integrative method in future work. First, instead of using binary TF binding profiles (binding/non-binding), incorporating quantitative profiles restricted by chromatin-mediated mechanisms, such as count of TF binding sites filtered by DNase-seq data, will provide more precise and valuable features. Second, TFs are multifunctional and typically cooperate to activate or repress genes, exerting a more complicated effect on transcriptional regulation than the assumption of a simple linear sum of TF activity. Accounting for combinatorial regulation in the model will greatly improve the method. Next, although this study focuses on a dysregulated transcriptional program, the method can be easily extended to incorporate other regulation. We found some residuals that seem to be higher around the fitted value of 0, leading to a slightly heavy tail of residue distributions (Additional file 2; skewness = 0.12, kurtosis = 1.26). One possible reason is that there are missing variables in the model. There are 22 genes with stage-dependent expression alterations that cannot be explained by CNV, altered methylation, or dysregulated transcription, which suggests that other regulation programs responsible for expression alterations in the late stage are not included in the model. TFs are one potential source of missing variables since only 206 TFs with known binding targets are included in the model compared with more than 1,000 TFs in humans [63]. MicroRNA (miRNA)-mediated post-transcriptional regulation is another possible source. Incorporating more TF binding information and miRNA expression profiles with sequence-based miRNA target information might further expand our knowledge of the underlying mechanisms of cancer progression. Another reason is that we only focus on stage-dependent changes in the model, so other factors that lead to expression alterations cannot be captured.

With the large-scale quantification of proteins becoming possible, the integrative method can be modified to model protein expression alterations beyond mRNA expression alterations to reveal potential translational or post-translational regulators that lead to protein abundance changes during cancer progression. Finally, this method is broadly applicable to any cohort of cancer patients for which copy number, methylation, and RNA expression profiles are known. Analyzing dysregulated transcriptional programs across all types of cancers simultaneously will provide the opportunity to explore whether there are common or specific regulators underlying cancer progression.

## Conclusions

Integrating copy numbers, methylation, and TF activity signatures to explain stage-dependent expression alterations presents a precise and comprehensive view on the underlying mechanisms during KIRC progression.

## Additional files

Additional file 1:
**Quality control of 178 samples.**
Additional file 2:
**Residual plots, histogram plot and normal Q-Q plot.**
Additional file 3:
**Comparison of our methods with lasso and ridge regression.**
Additional file 4:
**Significantly up-regulated and down-regulated genes in stage IV versus stage I KIRC.**
Additional file 5:
**Correlation of CNV and mRNA expression.**
Additional file 6:
**Correlation of methylation and mRNA expression.**
Additional file 7:
**Inferred activity changes of transcription factors in stage IV versus stage I.**
Additional file 8:
**Comparison of stage-dependent expression changes between TF targets and non-targets in the GSE36895 dataset.**
Additional file 9:
**The contribution of CNV, methylation and TF activity alterations to differential expression changes.**

## Abbreviations

CNV: copy number variation
FC: fold change
FDR: false discovery rate
GSEA: gene set enrichment analysis
KEGG: Kyoto Encyclopedia of Genes and Genomes
KIRC: kidney renal clear cell carcinoma
miRNA: microRNA
TCGA: The Cancer Genome Atlas
TF: transcription factor
