# Supplementary figures and images for: Transcriptome-wide signatures of tumor stage in kidney renal clear cell carcinoma: connecting copy number variation, methylation and transcription factor activity
Source: Genome Med. 2014 Dec 11;6(12):117. doi: 10.1186/s13073-014-0117-z (PMC4293006; doi:10.1186/s13073-014-0117-z)

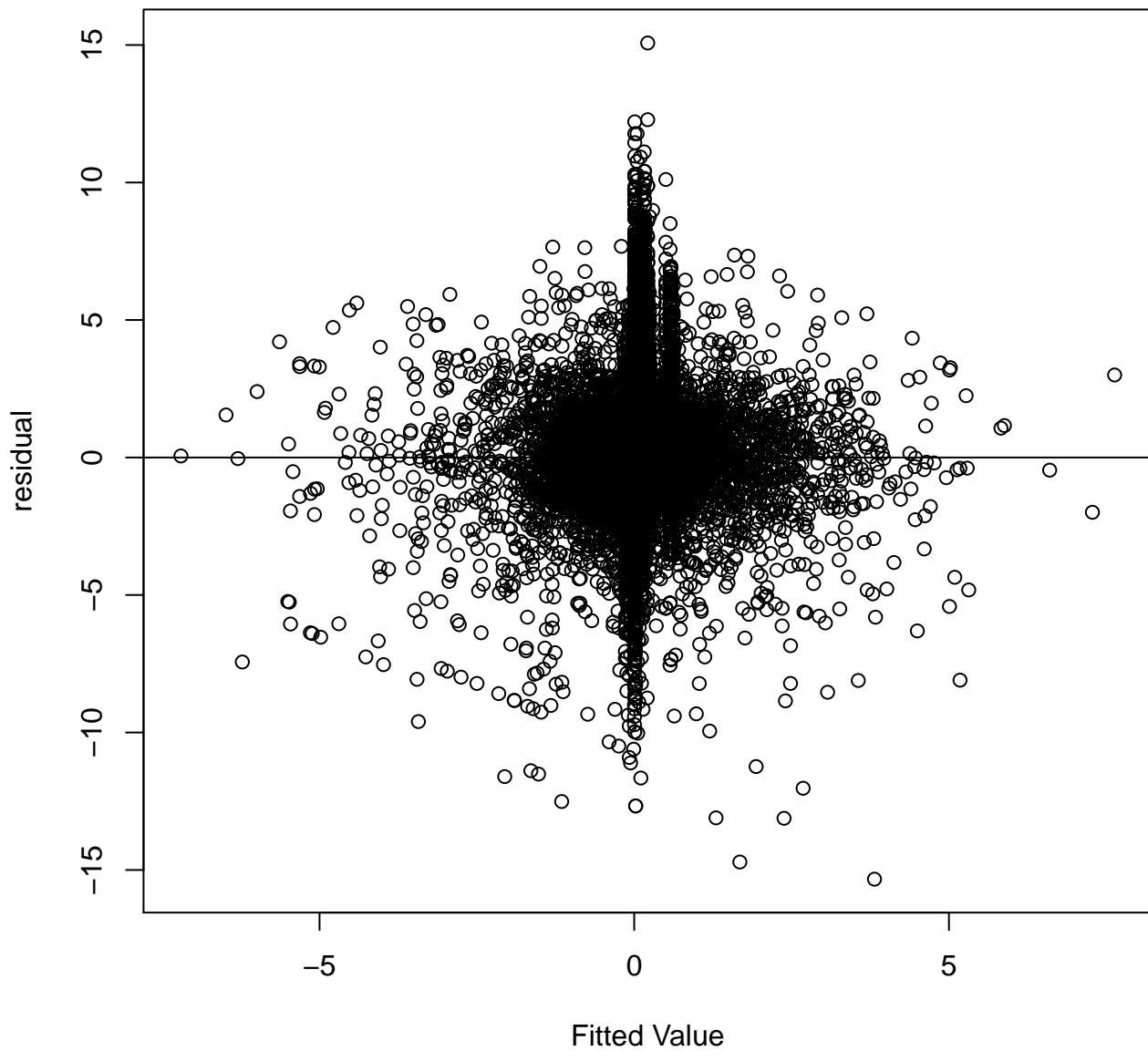

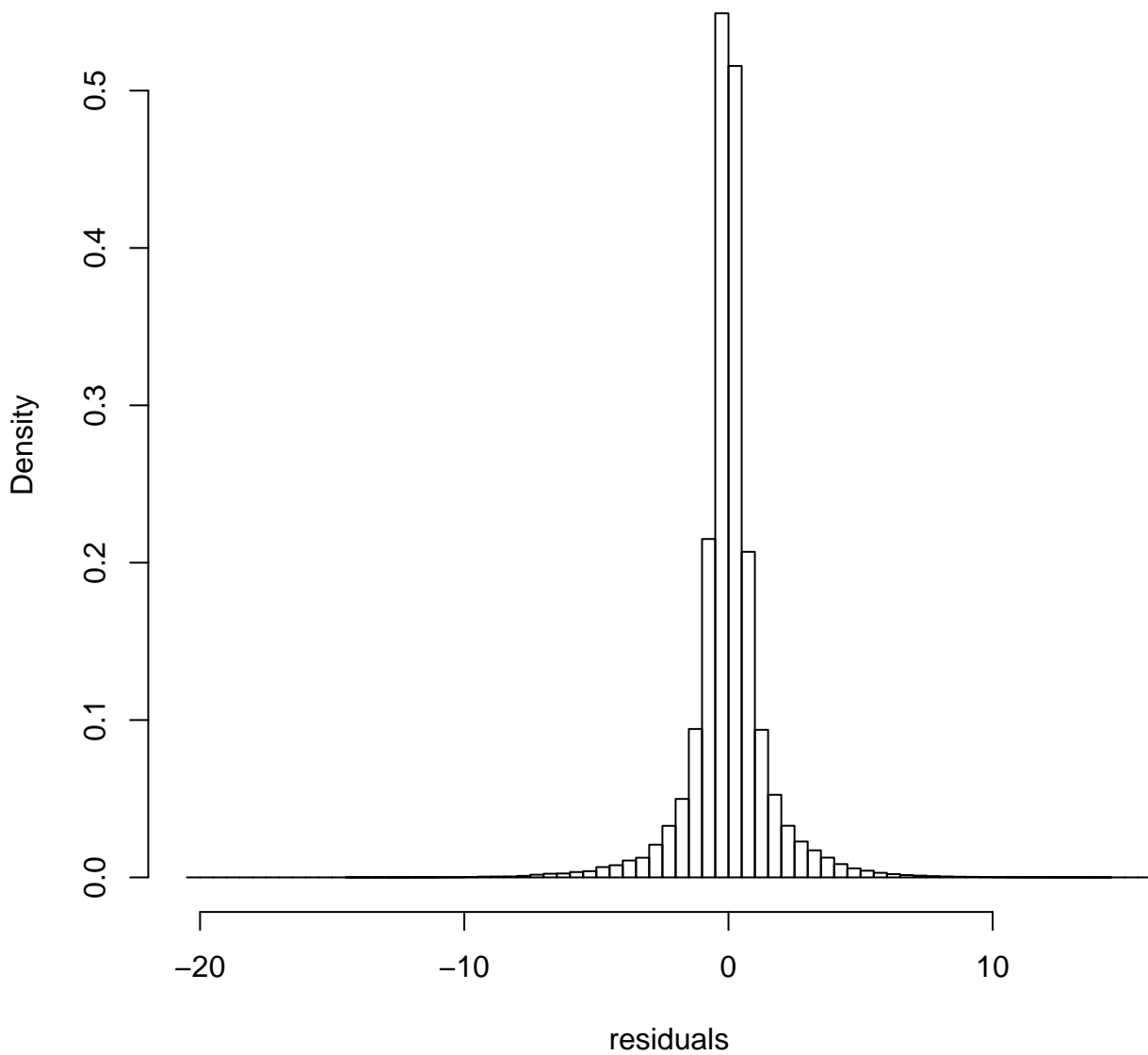

Normal Q-Q Plot

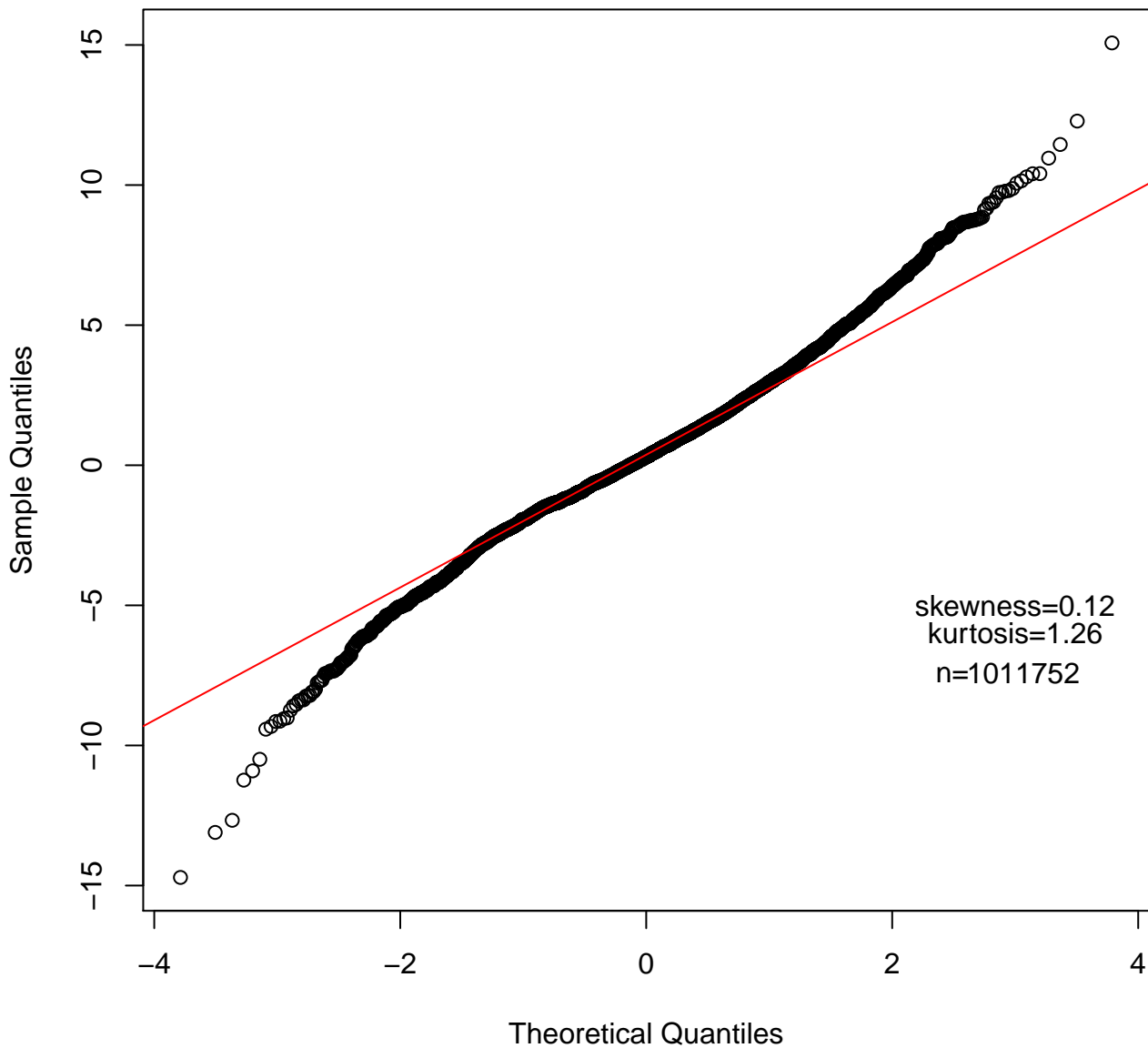

Supplement: Additional file 2: — Residual plots, histogram plot and normal Q-Q plot. [file 13073_2014_117_MOESM2_ESM.pdf]
