# Supplementary material for: Transcriptome-wide signatures of tumor stage in kidney renal clear cell carcinoma: connecting copy number variation, methylation and transcription factor activity
Source: Genome Med. 2014 Dec 11;6(12):117. doi: 10.1186/s13073-014-0117-z (PMC4293006; doi:10.1186/s13073-014-0117-z)
Supplement: Additional file 3: — Comparison of our methods with lasso and ridge regression. [file 13073_2014_117_MOESM3_ESM.pdf]

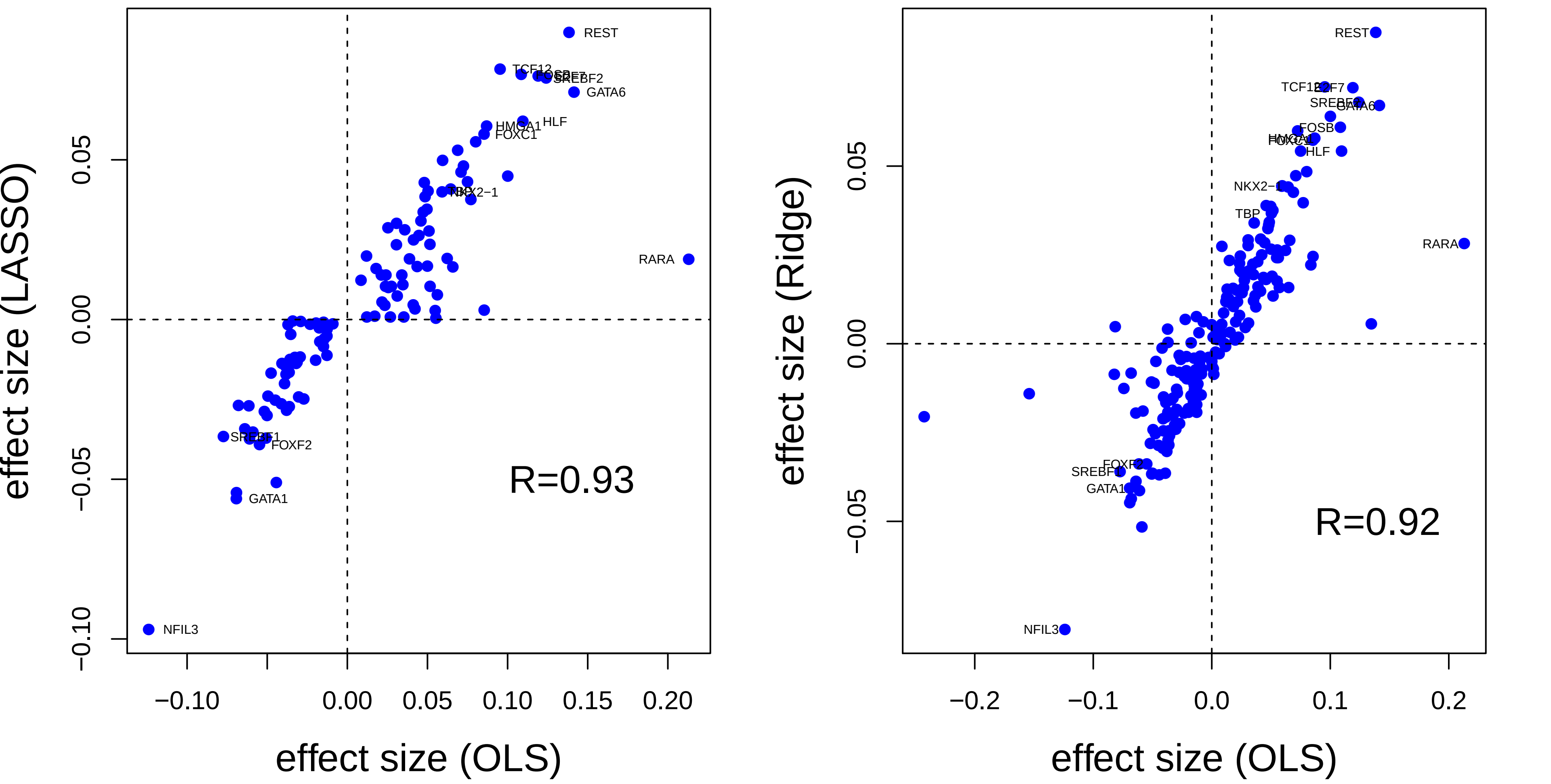

REST
TCF12
SREBF2
GATA6
HMGA1
HLF
FOXC1
NKX2−1
RARA
SREBF1
FOXF2
GATA1
NFIL3
R=0.93
effect size (LASSO)
effect size (OLS)
−0.10
−0.05
0.00
0.05
0.10
0.15
0.20
REST
TCF12
SREBF2
GATA6
FOSB
HLF
NKX2−1
TBP
RARA
FOXF2
SREBF1
GATA1
NFIL3
R=0.92
effect size (Ridge)
effect size (OLS)
−0.2
−0.1
0.0
0.1
0.2
−0.05
0.00
0.05

Effect sizes of the 16 TFs by our method (OLS), lasso and ridge regression

	OLS	Lasso		Ridge	
Regulator	Effect size	Effect size	Rank	Effect size	Rank
GATA6	0.14	0.07	7	0.07	6
NFIL3	-0.12	-0.10	1	-0.08	2
SREBF2	0.12	0.08	6	0.07	5
SREBF1	-0.08	-0.04	30	-0.04	35
TBP	0.05	0.04	23	0.04	31
HLF	0.11	0.06	8	0.05	12
TCF12	0.10	0.08	3	0.07	3
GATA1	-0.07	-0.06	11	-0.04	24
FOSB	0.10	0.08	4	0.07	8
RARA/RARB/RARG/RXRB	0.21	0.019	59	0.03	51
REST	0.14	0.09	2	0.09	1
FOXF2	-0.05	-0.04	25	-0.03	39
FOXC1	0.09	0.06	10	0.06	11
HMGA1	0.09	0.06	9	0.06	10
E2F7	0.12	0.08	5	0.07	4
NKX2-1	0.06	0.04	24	0.04	19
