# Supplementary material for: Transcriptome-wide signatures of tumor stage in kidney renal clear cell carcinoma: connecting copy number variation, methylation and transcription factor activity
Source: Genome Med. 2014 Dec 11;6(12):117. doi: 10.1186/s13073-014-0117-z (PMC4293006; doi:10.1186/s13073-014-0117-z)
Supplement: Additional file 8: — Comparison of stage-dependent expression changes between TF targets and non-targets in the GSE36895 dataset. [file 13073_2014_117_MOESM8_ESM.pdf]

One-sided KS test between stage-dependent expression changes of TF targets and non-targets

Regulator	p-value
**GATA6**	1.02E-06
**NFIL3**	8.38E-07
**SREBF2**	0.019502
**SREBF1**	4.68E-06
**TBP**	4.60E-12
**HLF**	7.48E-07
**TCF12**	0.879374
**GATA1**	5.49E-10
**FOSB**	0.00014
**RARA**	2.04E-07
**REST**	0.667569
**FOXF2**	4.68E-10
**FOXC1**	2.58E-05
**HMGA1**	0.019388
**E2F7**	0.069374
**NKX2-1**	0.001666
