# Supplementary material for: Transcriptome-wide signatures of tumor stage in kidney renal clear cell carcinoma: connecting copy number variation, methylation and transcription factor activity
Source: Genome Med. 2014 Dec 11;6(12):117. doi: 10.1186/s13073-014-0117-z (PMC4293006; doi:10.1186/s13073-014-0117-z)

Fig. S1 Expression similarity of stage I patients. mRNA expression profiles of TCGA-AK-3440, TCGA-B0-5098 and TCGA-B0-5117 are quite different from other samples, but all of them are composed of 85% tumor nuclei and less than 5% normal cells.

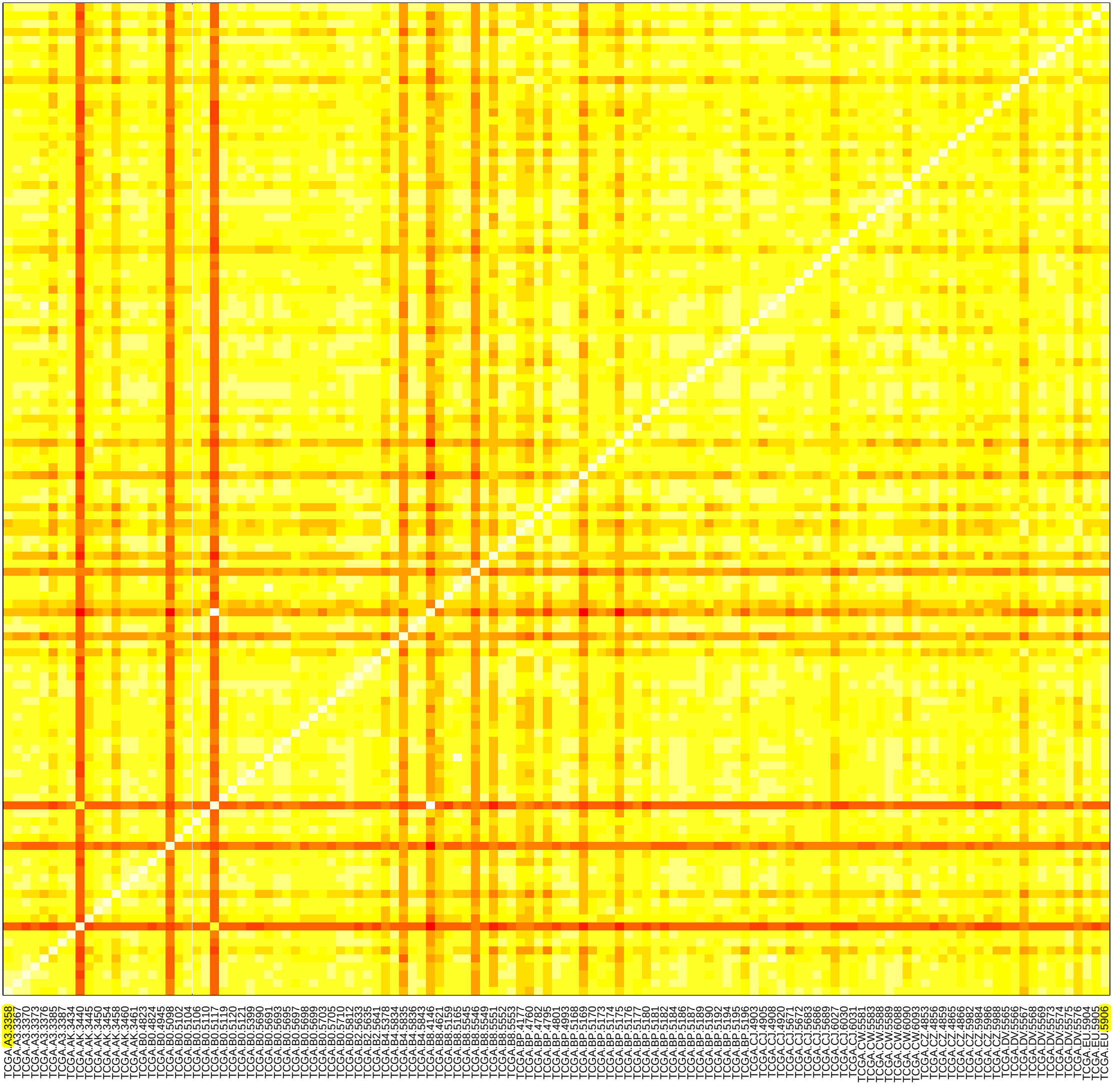

Fig. S2 Expression similarity between stage IV patients. Although TCGA-B0-4688, TCGA-B0-4698, TCGA-B0-4699, TCGA-BP-4770 and TCGA-CJ-5681 show different expression profiles from others, all of them are composed of at least 85% tumor nuclei and less than 15% necrotic cells.

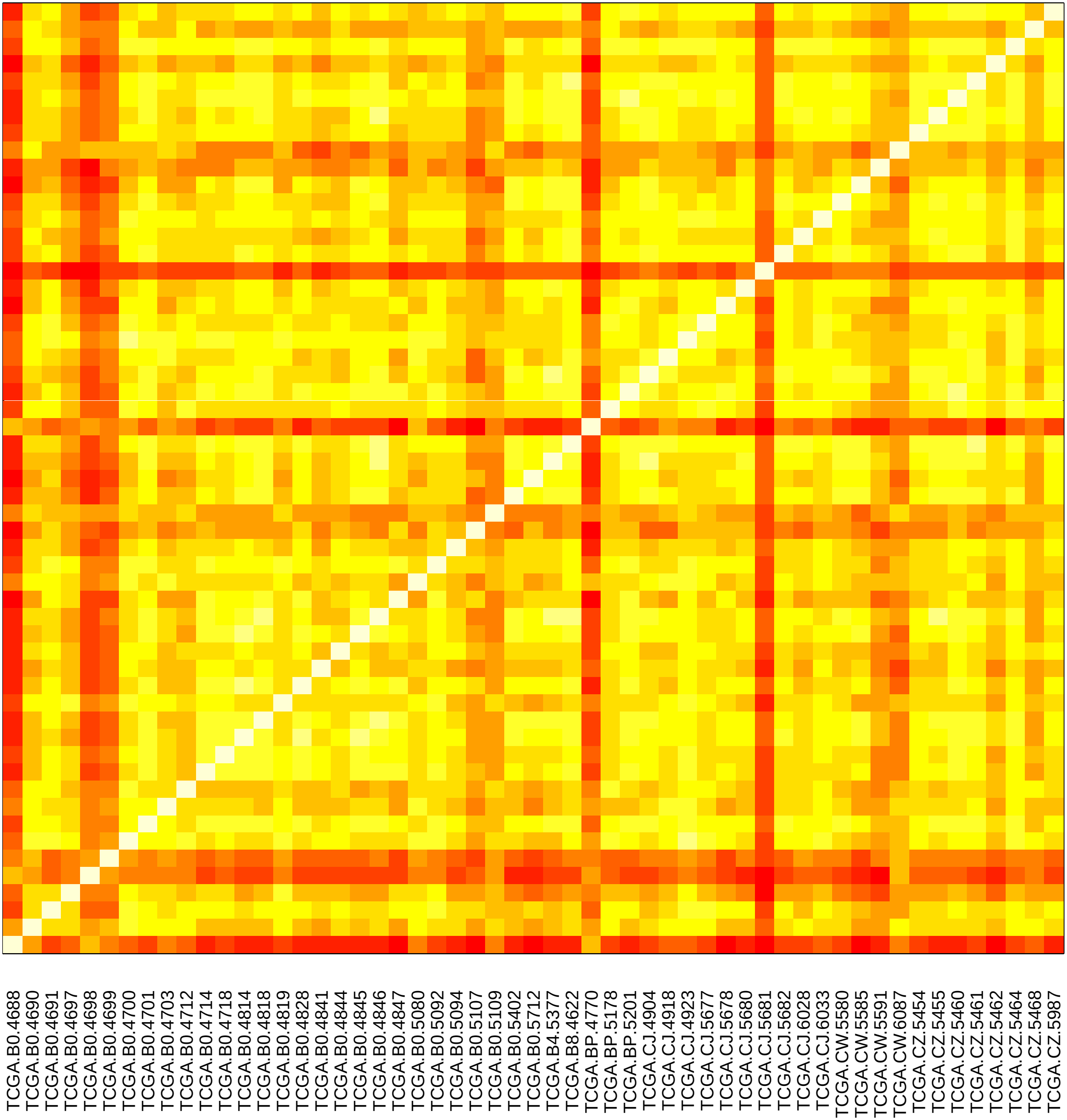

Supplement: Additional file 1: — Quality control of 178 samples. [file 13073_2014_117_MOESM1_ESM.pdf]
